# Promotion of planetary health through climate-sensitive health counselling in occupational therapy—a qualitative interview study in three countries

**DOI:** 10.3389/fpubh.2026.1826216

**Published:** 2026-05-07

**Authors:** Svenja Jeschonnek, Anna Christina Nowak, Annabell Duda

**Affiliations:** School of Public Health, Bielefeld University, Bielefeld, Germany

**Keywords:** climate change, climate-sensitive health counselling, health professionals, health promotion, occupational therapy, planetary health

## Abstract

**Introduction:**

Occupational therapy and planetary health are closely linked, as human health is influenced by environmental factors such as climate change. Occupational therapists (OTs) can enhance clients’ understanding of climate-related health issues and advocate for health-promoting climate action through climate-sensitive health counselling (CSHC). While CSHC has mainly been studied in medical contexts, its implementation in occupational therapy remains unclear. This study explores European experts’ perspectives on integrating CSHC into occupational therapy practice.

**Materials and methods:**

Six semi-structured interviews were conducted with occupational therapy experts from France, Germany, and the UK. Data were analyzed using content-structuring qualitative content analysis according to Kuckartz (2018).

**Results:**

Experts identified healthy and sustainable lifestyles and co-benefits of climate and health as key topics of CSHC in occupational therapy. CSHC can be integrated into routine healthcare and delivered within established therapy settings as well as settings involving well populations. Barriers and facilitators, including ethical tensions between individual and collective health perspectives and the availability of professional networks and movements, influence its implementation.

**Discussion:**

CSHC in occupational therapy can contribute to planetary health by supporting health protection and promotion. However, broader planetary health dimensions, such as human systems and environmental threats beyond climate change, are not yet sufficiently addressed. European perspectives and international collaboration may support the further development and implementation of CSHC in occupational therapy.

## 1 Introduction

The World Health Organization’s understanding of environment and health has been in place for decades, but increasingly profound human-induced changes to natural earth systems and global ecosystems are having negative impacts on public health, wellbeing, and equity for people worldwide (1–3). The comprehensive planetary health concept considers global ecosystems, human-made political, economic, and social systems, and health in an integrated way and is closely linked to finding solutions addressing environmental systems (3–6).

For populations in Europe, climate change is expected to lead to, for example, mental and heat-related health impacts and infectious diseases, with trends that are multidimensional and accelerating (7, 8). As a result, European healthcare systems and their professionals can expect to see more patients with climate-related illnesses such as cardiovascular diseases or injuries from extreme weather events (9).

One way in which planetary health can be reflected is in climate-sensitive health counselling (CSHC) (10). According to the pioneering scoping review by Quitmann et al. (11), CSHC refers to conversations between health professionals and their patients about climate change and its health impacts. It describes the integration of communication with patients on climate change into the health counselling of health professionals during a clinical visit or medical treatment (11).

The three overarching objectives of CSHC are to protect and promote individual and public health, to advance knowledge about climate change and health, as well as the awareness of patients, and to advocate for climate action and lifestyle change (11). The CSHC topics are the health impacts of climate change, healthy and sustainable lifestyles, and climate action, adaptation, and policies (11). Different health counseling communication strategies are used for the CSHC, for example, patient-centered communication, including a sensitive communication towards patients’ values and situations, active listening, shared decision-making, and motivational interviewing (11). In addition, climate change communication strategies include emphasizing climate and health co-benefits, linking climate change to patients’ individual health, providing space to discuss patients’ feelings related to climate change, and transforming climate anxiety into action (11).

The scoping review by Quitmann et al. (11) shows, on the one hand, a lack of European studies on CSHC, and, on the other hand, that, to date, no health professionals other than physicians have been examined. Occupational therapy as part of the healthcare system contributes to both a share of the mentioned burdens and tasks. Human occupations and activities are affected by environmental changes, and at the same time, they are in turn causing environmental changes, including human-made climate change (12, 13). Climate change negatively impacts the health and occupational performance of clients in occupational therapy, because they often have pre-existing health issues and are therefore at least part of vulnerable groups (8, 14, 15). For occupational therapists (OTs), climate change is experienced at personal, clinical, and professional levels. This includes, for example, addressing participants’ climate anxiety relating to personal experiences with extreme weather events, the lack of guidance and support structures, and the involvement of OTs in disaster management (16).

It is completely unclear to what extent CSHC is integrated into occupational therapy practice, although the profession plays an important role in healthcare and has a great potential to advocate for more sustainable lifestyles (17). Accordingly, this study explores an overview perspective from European experts on how CSHC could be implemented in occupational therapy. For this purpose, the profile and implementation of CSHC in occupational therapy are identified, including strategies, opportunities, and barriers associated with the framework conditions and the professional conditions for CSHC. Focusing on France, Germany, and the UK, countries where occupational therapy is well established in the respective healthcare systems, the European evidence on CSHC aims to be improved.

## 2 Materials and methods

### 2.1 Qualitative study design

This study applies an explorative qualitative research design to gain a fundamental understanding of CSHC in occupational therapy within the context of planetary health (18). This enables to gain a fundamental and exploratory understanding of the research subject, and to answer the open research question and its open sub-questions. Additionally, a qualitative approach leads to a multifaceted impression of the different aspects that can be described in detail throughout the analysis, which helps to develop new theories and further research questions (19). The principles of openness and problem-orientation of qualitative research make it possible to recognize unexpected aspects and to better understand the possible problem context of CSHC and Planetary Health in occupational therapy, which is particularly beneficial for the sub-research question of opportunities and barriers. The qualitative principle of holism helps to identify the actual situation and practice of CSHC in occupational therapy by consolidating the functional areas and spheres of life of OTs that may be explained during the interviews (20). For this purpose, interviews with experts were conducted to capture the practical knowledge of OTs and to gather information on the current development of planetary health in occupational therapy care across different European countries.

Based on the theoretical background and the research questions, the semi-structured interview guideline was developed by relying on the methods and rules described by Döring and Bortz (19), Helfferich (21), and von Dem Berge (22). In detail, the guideline questions were developed by applying the approach of Helfferich (21): At first, various potential questions were collected, followed by examining these questions concerning the research interest. Then, the remaining questions were sorted into content-related question bundles and were put in chronological order. Finally, in-depth questions were subordinated under higher-level narrative-generating questions in question bundles. The final interview guideline comprises four thematic sections: (1) introduction of the participants and their work; (2) topics and aspects of CSHC; (3) situations, settings, and strategies and approaches; and (4) opportunities and barriers. After the first section, the participants were asked to refer exclusively to their respective country in the interview and to clearly indicate statements with international references. This should help generate insights into occupational therapy professions across different countries and distinguish them from one another.

The interview guide (English version) was tested in a pre-test with an OT from Germany. The final version was translated into German and French. All interviews were conducted by the first author of the paper, who is herself a speech therapist^1^ and therefore part of the allied health professions. German is the first author’s native language, and language skills in English and French range from proficient to fluent.

### 2.2 Recruiting experts and conducting interviews

As planetary health aspects (e.g., sustainability) are not yet specifically or broadly integrated into occupational therapy, occupational therapy experts should be selected for their specialized knowledge and insight in this area (23). An overarching national perspective on occupational therapy can be ensured by including experts who are members of national professional organizations, networks, movements, or academia. This means that the experts are not only doing clinical work. All inclusion and exclusion criteria are listed in Table 1. Different country perspectives are to be considered, so that experts with merely an international perspective or who work are excluded as well.

**Table 1:** Recruitment inclusion and exclusion criteria.

Inclusion criteria	Exclusion criteria
Obtaining a degree in occupational therapy	
Working in a European country	Only working on international levels/with a merely international perspective
Having overarching insights into the occupational therapy profession on a national level in a European country	Doing exclusively clinical work in occupational therapy with no overarching professional insights on a national level
Special knowledge about planetary health in occupational therapy	
Language skills in English, French, or German	

Potential experts were identified based on identified narrative, descriptive, and commentary literature, as well as identified networks and movements. This revealed suitable experts from Germany, France, Sweden, and the UK who have been requested via e-mail and, in one case, via recommendation from another recruited participant. In addition, a detailed research advertisement has been published on the website of the World Federation of Occupational Therapists (WFOT), and multiple networks and movements have been contacted. In total, 10 individual experts have been contacted directly, two of them without reply, two refusing (one of them without giving reasons, one due to unfamiliarity with the specific term of CSHC). Despite the call for participants on the WFOT website, no one has proactively contacted the authors. Based on the replies, the study focused on Germany, France, and the UK.

Finally, six participants were recruited, two each from Germany, France, and the UK. The sample size was determined based on principles of qualitative research rather than statistical representativeness. Given the narrow research aim and the relatively specific and homogeneous sample of occupational therapists, a smaller sample size was considered appropriate, in line with the concept of information power (24). Additionally, the study addresses an emerging topic within occupational therapy, which contributed to challenges in recruitment and further highlights the exploratory nature of this research. All interviews were conducted and later analyzed in the interviewee’s respective native language.

### 2.3 Conducting and analyzing the interviews

All interviews were conducted via Zoom. The transcription of the interview records was carried out by using f4x, an automatic audio recognition program. The generated transcripts were edited manually using the simple transcription rules for computer-assisted analysis according to Kuckartz (25). Anonymization of individual names, organizations, networks, and movements was part of the transcription to prevent the identification of the interviewees. MAXQDA 2022 was used to edit and analyze the transcripts. Coding was conducted by the first author.

Based on the content-structuring qualitative content analysis according to Kuckartz (25), three main categories were deductively defined: *CSHC profile*, *CSHC implementation*, and *opportunities and barriers*. For the last two, subcategories emerged inductively.

In a final analysis step, an iterative process was conducted. In this process, the main categories were evaluated on a category basis, and connections between sub-categories within the main categories were considered. In the analysis and presentation of results and discussion, the respective country reference is considered and presented as statements about the respective national occupational therapy professional group.

### 2.4 Researcher reflexivity and ethical considerations

Our research team conducted this exploratory interview study on climate-sensitive health counselling in occupational therapy from a public health perspective. This disciplinary positioning influenced both the formulation of our research aim and the interpretation of the interview data. The first author contributed a therapeutic perspective through her background as a speech and language therapist, broadening our understanding of the relevance of climate-sensitive counselling across therapeutic professions. Additionally, the other two authors contributed extensive experience in qualitative research, facilitating a reflective team process throughout the generation and analysis of data. We remained attentive to how our professional backgrounds, assumptions, and prior expertise may have influenced the research process and our interpretation of the findings.

The study has obtained a favorable ethical approval from the Ethics Committee of Bielefeld University (application No. 2023-137 of 2023/06/09). The study was rated as uncritical by the ethics committee of Bielefeld University, which means it was considered to pose no foreseeable ethical risks to participants and therefore did not require full ethical review.

## 3 Results

### 3.1 Overview of the interviewed experts

The interviews took place in July 2023. The duration of the interviews ranged from 0:57 h to 1:19 h. Three experts are currently not working in clinical occupational therapy practice. One works in clinical occupational therapy at weekends for a mental health crisis intervention team. Another one is clinically active in the field of refugees’ mental health. One expert works in urgent care management for an occupational therapy service and a community project. The participants’ connections to planetary health are multifaceted. All participants, except one, have published articles or documents on occupational therapy and, in a broader sense of planetary health, e.g., in the field of sustainability and health, which are not specified due to anonymization. All participants have an overview perspective on occupational therapy in their respective countries through their involvement in networks, their professional activities, and/or their publishing activities (see Table 2 for details).

**Table 2:** Overview of participating experts.

Participant	Gender	Country	Additional studies	Current role	Clinical activity	Planetary health connection paired with national overview perspective on occupational therapy
P1_GER	Female	Germany	Environmental science	Teaching	No	Teaching, civil society movement on health and climate change, national professional association of occupational therapy
P2_FR	Female	France	/	Teaching coordinator	Formerly	National health and sustainability network in occupational therapy
P3_FR	Male	France	Health science education, health sociology	Research and development (AT company)	Formerly	Entrepreneurship sustainable AT, former teaching, former member of a national health and sustainability network in occupational therapy
P4_UK	Male	UK	/	Strategic advisor	Yes	Strategic and advisory centre for sustainability in healthcare, international projects
P5_GER	Female	Germany	/	Clinical OT	Yes	Civil society movement on health and climate change
P6_UK	Female	UK	/	Urgent care management, community project	Yes	Network of sustainability in occupational therapy

### 3.2 CSHC profile

All experts define CSHC as talking, informing, and giving advice about climate change-related and environmental health impacts, and how to improve clients’ health. CSHC in occupational therapy is about OTs sensitively, in a client-centered way, introducing and holding conversations with clients about the ecological and climate crisis, awareness, anxiety, concern, and feelings, in a therapeutic context of healthcare. One participant from Germany suggests expanding the term of CSHC to “planetary health counselling.”

*“Climate-sensitive health counselling or in general planetary health counselling - I think I would prefer that term.” (Translation, P1_GER).*

All participants mention objectives of CSHC regarding the clients: prevention and health promotion, encouraging climate co-benefits, promoting climate and environmentally friendly or sustainable occupations and occupational justice, creating or improving knowledge and awareness about the climate change and environmental crises, their (health) impacts, people’s own dismay and responsibility, and supporting, showing, and talking about people’s options to act on climate change. Associated objectives are reducing potential climate anxiety and potential emotional defenses or vigorous objections against the topic of climate change, multiplying the topic of climate change and health in society, satisfying clients’ needs regarding health and security, and shifting the focus of clients regarding climate mitigation from the environmental footprint to the handprint.^2^

All participants present topics and aspects of the CSHC by OTs. They mention recycling and waste reduction, for example, occupational therapy’s assistive technology (AT), physical activity and active mobility (such as walking and cycling), nutrition, contact with nature, environmental impacts of medicines, housing, and sustainable jobs.

*“One can consider that also the promotion of physical activity is a sustainable recommendation, in a certain way […], it is promoting active mobility, for example, instead of motorized transport […]. And what develops, but is still marginal, is the whole counselling around nature contact (.), and the benefits that nature-based occupation can bring.” (Translation, P2_FR).*

Climate mitigation and adaptation measures in their broader sense can be topics of the CSHC, as well as the fields of action mentioned in the Guiding Principles for Sustainability of the WFOT. Daily occupations and their climate and health co-benefits can represent topics of CSHC. Similarly, topics can arise from the individual client, such as potential (non-)communicable health impacts of climate change.

### 3.3 CSHC implementation

#### 3.3.1 Settings and situations

CSHC can happen in multiple settings. These can be established in therapy settings within medical and social facilities, in health promotion and prevention settings, in local living environments or communities, or in preventive and health-promoting settings. This means that clients in need of healthcare interventions, as well as healthy populations without specific treatment needs, must be addressed. Five of six participants mentioned nature as a setting, especially in mental health contexts. OTs in the UK are already aware of the sustainable value of accessing green spaces, for example, with the approach of nature walks and talks. One-on-one as well as group settings can be suitable for CSHC. In all cases, settings should be non-accusatory for the clients.

*“The question is to what extent this is really something additional or to what extent it simply flows into what we are already doing? We already offer counselling services.” (Translation, P1_GER).*

All participants mentioned everyday situations for implementing CSHC. These include, for example, general counselling in occupational therapy, shopping, cooking, and eating, improving active mobility, emphasizing an already engaged urban environment, discussing equipment in occupational therapy, and managing the client’s home. Furthermore, the five participants who mentioned suitable situations described situations that are individually adapted to clients and consider their life circumstances, occupations, affectedness, and interests or openness.

*“But (.) it is also important to be as close as possible to people’s life circumstances. (.) And occupational therapists are well placed to do this. (.) They can create scenarios, they can make home visits, (.) they know how to observe occupations and activities.” (Translation, P2_FR).*

#### 3.3.2 Approaches and strategies

Strategies for including CSHC in OT practice include engaging in physical activity or nature-based activities, incorporating global ecosystems into OT activity analysis, and building on the therapeutic relationship. It was a consensus that CSHC can be implemented in therapeutic practice.

*“Yeah, it’s this (.) think globally, act locally. So, I think what needs to happen at the core is incorporating the global ecosystems into our activity analysis. (.) So I do not think that will change (.) our interventions so much. But it will change (.) our engagement with them and the conversations that we have. “(Original, P4_UK).*

The client- and occupation-centered approaches included providing or developing together with the client individually appropriate and feasible options for actions, and promoting environmentally friendly or sustainable occupations and interventions, for example, by offering low-carbon treatment options.

*“Thinking about eating mindfully, and really engaging with it, and think about where the food comes from. And that can (.) at occupational purpose, occupational meaning (.), so engaging with this on a deeper level can have wellbeing, lots of wellbeing effects. […] Hopefully, the engagement will give people more occupational meaning, more occupational purpose, and improve their well-being.” (Original, P4_UK).*

Client- or community-centered communication was mentioned by the German and British participants. These are, for example, putting benefits for the client or communities first, keeping the CSHC informative and attractive, and applying professional communication methods, such as motivational interviewing. Communication in CSHC could be non-verbal or verbal, including written communication (e.g., flyers and posters).

Possible indirect strategies and approaches for CSHC are, for example, offering (more) sustainable occupations, or using nature connections, while an example of a direct strategy is actively addressing sustainability issues while working with clients on healthcare goals. Further strategies and approaches of the CSHC are pointing out possible ways of reducing one’s footprint and improving one’s handprint, and opportunities on the structural level of informing and influencing others to act on climate change, empowerment, and educational methods.

All participants mentioned that client-centered strategies of CSHC should be applied sensitively. This includes to respect and to consider the clients’ perspectives and conditions to get involved in CSHC, as their awareness about environmental issues and their capacity and willingness to talk about and actively engage with them, while considering their socio-economic status, biography, age, social norms, as well as individually established habits, environmental efforts already made, and one’s emotional state. It needs to be recognized how climate change affects the clients, including their (non) responsibility, resources, vulnerability, awareness, and motivation to act on climate change.

*“And I think sensitive is the really key word there because it’s not for us to (.) use (.) our advantage and our professional place to put such a worry onto vulnerable people or something like that.” (Original, P6_UK).*

### 3.4 Opportunities and barriers

#### 3.4.1 Framework conditions

The examined countries offer their populations many opportunities that allow for individual behavioral changes, and at the same time, the people of these high-income countries consume a lot of resources, which harms the environment. People and healthcare systems, reflecting the carbon and waste intensities of the societies, increasingly care about greenhouse gas emissions. For example, P4_UK mentions that in Wales (UK), a new future generations legislation is in force, which focuses on sustainable development for the healthcare system. In Germany, the interviewees mention that the issue of climate change is very present in the media, and there is a growing municipal commitment to climate action. At the same time, the participants declare Germany’s and the UK’s current national policy frameworks as insufficient for environmental protection and planetary health. For France, a question named by P2_FR is, how to achieve policies in terms of socially, environmentally responsible and planetary health, positively influencing decisions.

Regarding climate change as a framework condition itself, the participants observe noticeable changes in the environment with an influence on human behavior, e.g., choice of residence in later life and rising climate change-induced migration. On the one hand, health-influencing factors are changing, which may be challenging to deal with as an OT. On the other hand, the impacts of climate change do not seem to be sufficiently noticeable to everyone.

In terms of working conditions, a facilitator is that clients in France can access occupational therapy free of cost, including potentially needed AT. There are also increasing numbers of health-promoting programs mentioned by the participants, and OTs in France have the opportunity to develop new forms of practice by themselves. In Germany, a relatively large amount of time is spent with each client, which can strengthen a trusting relationship between an OT and the client. Additionally, according to P5_GER the possibility of domiciliary could be helpful. According to the participants, UK is offering “green prescribing” in occupational therapy, but also outside of that, it is important to consider the natural and built environment of the respective institution of an OT, which, for example, does or does not provide green spaces.

#### 3.4.2 Professional conditions

A lack of time is described as a working-condition issue for OTs in the UK and Germany in relation to the implementation of CSHC. In Germany, there is no established community-based practice yet. In France, clients could access occupational therapy only through a prescribing physician, who typically adopts a biomedical approach and therefore does not refer clients to OTs for environmental reasons. Furthermore, the role of French OTs in planetary health remains unestablished.

Various resources indirectly support OTs in advocating for planetary health: the (international) Guiding Principles for Sustainability of the WFOT, which also includes a German translation, and the WFOT Code of Ethics. There were also national resources, such as the guidelines of the German National Association of Occupational Therapy, the UK’s Greener NHS, the sustainability agenda, professional standards, and a program of sustainability and quality improvement. Different planetary health information material, literature, and events are available in the UK and Germany, for example, workshops about the WFOT Guiding Principles (representing an implicit integration of planetary health and CSHC, and an ethical encouragement). In France, the national association of occupational therapy holds conferences on the socio-ecological transformation.

The importance of education is mentioned by five of the six participants. For example, integrating knowledge about and recommendations for addressing the relationship between climate change and occupational therapy into advanced training courses for OTs could improve OTs’ knowledge and skills in advocating for planetary health, including skills to support behavior change and knowledge of relevant resources. Education encompassed initial training as well as further or advanced training.

The need to improve the exchange among OTs and the important role of movements and networks are expressed by five of six participants. These include grassroots civil society movements for climate change and health in Germany, and sustainability networks and working groups for OTs in France and the UK. Movements and associations can promote and advocate for the topics of planetary health in society and in the occupational therapy profession.

*“But I think, the network only developed like that because the connection with other countries was established right from the start. It is in contact with Quebec, Switzerland, and Belgium.” (Translation, P2_FR).*

As well as networks and working groups, they could promote networking, engagement, and exchange on planetary health. Networks could support OTs to pursue their values not only in their private life, which was expressed to be easier, but also in their professional life. They could enable and encourage OTs to talk about planetary health and the changes they can make in the healthcare system.

The participants mentioned different states of interest and awareness among OTs in the different countries. In Germany, many OTs attend congresses and workshops about or including planetary health.

*“I think that it is promoted in occupational therapy, and that many [OTs] are more interested in that, simply because the environment always plays a role [in occupational therapy].” (Translation, P5_GER).*

Even if there is little time for planetary health in the everyday work of OTs in Germany, a basic understanding of its topics among OTs in Germany seems to be prevalent, with positive and skeptical attitudes towards conducting CSHC being noticed. For the UK, the situation was unclear. Many OTs are interested in sustainability issues, but there is also a lack of awareness of planetary health.

*“I’ve met lots of occupational therapists who are interested in sustainability issues, green issues in their own personal lives. But they think that when they get to work, it has not to do with them.” (Original, P4_UK).*

*“I think what’s more challenging is (.) actually the lack of awareness (.)” (Original, P6_UK).*

In France, OTs are aware of the environmental impact of AT, as they seem to prefer second-hand over new tools, they are interested in nature-based interventions, and they see the potential in the connection between the environment, health, and occupations. The French participants also recognized a sensitivity, awareness, and openness of many OTs towards planetary health topics.

*“[…] in France, in general, (.) the therapists are very sensitive towards the topic. (.) So, I think it is a value that is carried by many occupational therapists.” (Translation, P2_FR).*

In general, there are supporting values present in the profession, such as the voluntary of caring for others, and environmental values, but whose implementation in practice depends on the individual social environment and external conditions. At work, they see environmental responsibility not particularly by themselves. For instance, the participants estimate not many but increasing numbers of OTs in France, and many OTs in Germany, conducting the CSHC consciously and unconsciously. The practice of CSHC by OTs is still in its infancy and depends on the willingness, capabilities, initiative, and attitudes of the individual OTs, such as individual moral maturation and moral conation.

*“And, there are some that start to integrate it. But all that, I have to tell you, so, it is still (.) at beginner level […]” (Translation, P2_FR).*

OTs know about occupational determinants and change. But there is not enough knowledge about environmental topics and how to advocate for planetary health. Many German OTs probably already heard about planetary health in some articles, but in-depth knowledge is missing.

*“The knowledge, (.) the factual knowledge is not available. Everyone has to do that individually. I also do not believe that it is widely known in the broader professional field. This knowledge is simply from the connection (.) or planetary health in general. “(Translation, P5_GER).*

The potential role of OTs in CSHC was multifaceted. They could be seen as behavior change specialists and have a role in health promotion and prevention. As part of a multiplier role, a wide variety of clients could be reached in different areas of life and society.

*“And I think as occupational therapists we are behavior change specialists (…) and the occupational therapy process could be applied to anybody (.) in a well population who wants to live more sustainably.” (Original, P4_UK).*

But it is unlikely that OTs describe themselves as counsellors, even if they have a role of change agents. There are doubts among OTs about whether it is their role as health professionals to advocate for planetary health. Participants from France and Germany describe a conflict between the individual and collective ethical perspectives.

*“If one is presenting a sustainable recommendation, […] it may come into conflict a bit with this individually centered ethic. Because, as a result, one puts at stake another form of […] collective, community-centered ethic […]” (Translation, P2_FR).*

*“Can we justify this ethically or not to talk about it? I would say, I am at the point that it is not an on-top task, but something we need to consider because this influence is there” (Translation, P1_GER).*

One’s own position must not be abused, and the client’s individual decisions and values must be respected. There is an uncertainty as to whether OTs are ethically allowed to advocate for planetary health, but at the same time the present and observable health impacts of climate change need to be addressed as health promotion is an objective of occupational therapy, non-harmfulness an ethical principle, and the Guiding Principles for Sustainability of the WFOT allow or encourage OTs to integrate sustainability into their practice.

## 4 Discussion

The study aimed to explore a European expert’s overview perspective of how CSHC could be implemented in occupational therapy. For this purpose, the profile and implementation of CSHC in occupational therapy were identified, including strategies, opportunities, and barriers, thereby demonstrating the practice and potential of CSHC in occupational therapy. Six interviews were conducted with OTs from Germany, the UK, and France. We asked them about their understanding and the current practice of CSHC in occupational therapy and perceived opportunities and barriers.

The interviews suggest no clear definition of CSHC in occupational therapy. However, the expressed defining aspects of the interviewees suggest an overlap with the CSHC definition but also mainly with CSHC’s profile, including objectives as well as topics and aspects, described in the scoping review by Quitmann et al. (11). Objectives of CSHC in occupational therapy, such as improving clients’ knowledge and awareness as well as advocating for climate action, are mostly consistent with the objectives named by Quitmann et al. (11). The objective of lifestyle changes from Quitman et al. (11) is described by the participants from an occupational therapy perspective as promoting ecological/sustainable occupations, since occupations are part of lifestyle as they can be categorized as self-care, productivity, or leisure according to the definition by Creek and Lawson-Porter (26). Promoting health and climate co-benefits is in line with Quitman et al.’s (11) objective to protect and promote individual health. The co-benefits place the focus on health promotion with positive climate effects or climate protection with positive health effects (11, 27, 28). Topics of the CSHC in occupational therapy are healthy and sustainable lifestyles, e.g., in the areas of nutrition, physical activity/active mobility, and nature contact. Precisely these three areas of CSHC are also described by Quitman et al. (11) as frequently mentioned topics of synergies between healthy and climate-friendly lifestyles. Health impacts of climate change, especially in the sense of personal adaptation, were another frequently mentioned topic among the participants and coincides with the topic of health impacts and adaptation identified by Quitmann et al. (11). The third major topic—climate action and policies—mentioned by Quitmann et al. (11) is hardly found in the interviews. Addressing both health and climate co-benefits in CSHC in occupational therapy seems to be useful to pursue the objectives of CSHC and to improve climate mitigation and adaptation, as promoting the health co-benefits is part of health protection and promotion in the planetary health concept (3, 28). These topics and aspects supporting the objectives of CSHC in occupational therapy should be described in more detail in further research, and other potential topics and aspects should be identified.

Regarding the implementation of CSHC in occupational therapy, two overarching setting areas have been identified in the interviews: established therapy settings and with well populations. This includes community, group, and nature settings. The naming of nature settings is consistent with the naming of nature contact as a topic. According to Dieterle (12), contact with nature in occupational therapy can increase the motivation to protect it. In these settings, different situations of OTs’ everyday work can be implemented or represent starting points for the CSHC. Like dietitians and nutrition professionals, who apply planetary-health content across diverse practice settings and everyday professional routines, OTs can embed CSHC into familiar occupational situations rather than treating it as an entirely separate intervention domain (29). Often, the situations correspond to the previously mentioned topics of CSHC: cooking and eating situations overlap with the topic of nutrition. Situations of improving active mobility overlap with the topic of physical activity or active mobility, and so on. At the same time, situations of CSHC need to be sensitively adapted to the individual client. The concept of planetary health is also gaining increasing importance among other healthcare professionals, such as nurses. The review by Vandenberg et al. (30) underscores the need for action, the necessity of interprofessional collaboration. This broader perspective suggests that CSHC may be positioned not only as an intervention in selected occupational situations but also as part of a wider professional mandate to respond to planetary health challenges in collaboration with other health professions.

During the qualitative analysis, it was found that they are rather strategies and approaches than clearly defined or evidence-based methods. Some of the strategies mentioned by the participants can be easily linked to situations and topics; some can be used across topics and situations, such as incorporating global eco-systems in OT’s activity analysis, building on the therapeutic relationship, empowerment, and educational methods. Interventions do not have to be completely changed to integrate the CSHC. It is more of a change in the engagement with the situations and conversations that OTs already have with their clients. This underlines the integration of the CSHC into routine healthcare activities, which corresponds to Quitmann et al. (11), who identified this integration as an overarching principle. The mentioned approaches of client- and occupation-centeredness are consistent with this and at the same time major components of occupational therapy (31). In particular, occupation-centeredness in CSHC can address the close relationship between occupations and climate change, according to occupations representing both the cause and the solution of climate change (12, 13, 32). On the one hand, the client-centered approach can also be found in Quitmann et al. (11) as part of the health counselling strategies. On the other hand, client-centered approaches may support or even hinder the CSHC by OTs, depending on the values of the individual client and the extent of the biomedical focus of the OT (33). In addition, focusing on (co-)benefits for the client is part of the client-centered communication, and can be found in Quitmann et al. (11) as part of the climate change communications strategies, as well as in the scoping review of Smith et al. (32) on the role of occupational therapy in environmental sustainability. Indirect and direct approaches can both be found in client-centered and routine healthcare situations. On the one hand, indirect strategies of CSHC signify a client-centered approach as they do not collide with the client’s potentially different attitudes and values regarding climate change (11). On the other hand, not all objectives of CSHC, especially improving knowledge, can be pursued only indirectly (11). Sensitivity towards the client can be found in Quitmann et al. (11), where climate-sensitive means climate-related communication as well as sensitive communication. This aspect appears to be highly important in the CSHC by OTs. Furthermore, the participants partly reflect the evidence-based principles of communication on climate change and health according to Peters et al. (34). Further research is needed to identify specific and evidence-based methods that fit the described approaches and strategies. Additionally, research is needed to measure the actual effects of CSHC implementation, e.g., which effects have approaches, strategies, and methods in the described situations, to achieve the objectives of CSHC. According to the current evidence, effects of improved knowledge, but very limited to improved climate-friendly behaviors, have been observed (35).

Networks, movements, and national professional associations can provide resources and support for OTs to advocate for planetary health. Also, other mentioned barriers as a lack of time and unsustainable healthcare systems, can be fields to act on for the networks, movements, and professional associations. OTs’ education can be an opportunity to improve the prerequisites of CSHC, but, for instance, more integration of planetary health topics into education is needed, for which organizations can support the OTs as well. Specifically, there is a need for further informational and guiding material for planetary health advocacy developed especially for occupational therapy (36). Support on these different structural levels could be a lever for CSHC in occupational therapy.

Regarding the prerequisites of OTs in the three countries, they have varying degrees of interest, awareness, attitudes, values, and knowledge regarding planetary health. Therefore, it can be assumed that the planetary health literacy of OTs generally needs to be improved (37–39). OTs have communication, client-centered, preventive, and behavior change competencies that can help to conduct the CSHC. The role of OTs is occupation-oriented, health-promoting and preventive, client-oriented, or that of change agents. At the same time, barriers are coming from an ethical conflict between the individual (client-centered) and collective (public health-oriented) ethical perspectives on health in occupational therapy practice. The Code of Ethics of the WFOT can be guiding in this conflict (40): OTs are responsible towards the local and global society, and they promote health on local, regional, and global levels. In addition, a statement of the WFOT on climate change, the environment, and health, outlines that OTs advocate for sustainable occupations and address relationships between the environment, occupations, and people in order to promote engagement in needed or desired occupations (41). There are health impacts of climate change and other environmental threats for the clients why it is important to address these issues not only from a public health perspective but also from a client-centered perspective that is inherent to occupational therapy. Therefore, these health impacts should be part of the ethical and clinical reasoning of OTs and are not standing against the client-centered perspective of occupational therapy. Deepening and taking this ethical discussion is needed to overcome the willingness-action gap of many health professionals regarding climate and health communication and advocacy (11, 42).

European OTs could learn from the different countries’ expertise and insights, especially through international networks, prescription of nature-based interventions, subject-specific informational resources, and the approach of the environmental handprint. In general, improving cooperation in occupational therapy between European countries could help to exchange resources and experiences between countries, not only between France, Germany, and the UK, and not only for CSHC and OTs but also for planetary health advocacy and allied health professionals.

Future research should apply concepts from implementation science, such as the Consolidated Framework for Implementation Research (CFIR), to systematically identify barriers at various levels (individual, organizational, systemic) as well as factors that facilitate the integration of CSHC into occupational therapy practice. While our findings suggest promising settings and professional contexts as entry points, empirical studies are needed to jointly develop and test tailored implementation strategies, including stakeholder engagement, capacity building, and decision-support protocols, that ensure sustainable acceptance, adherence to guidelines, and the expansion of CSHC across various therapeutic contexts. Mixed-methods and hybrid effectiveness-implementation designs could evaluate not only CSHC effectiveness but also key implementation outcomes like acceptability, feasibility, and cost-effectiveness, particularly addressing common challenges such as overburdened practitioners, intersectoral collaboration deficits, and limited local ownership observed in climate-health adaptation efforts. Such research would bridge the evidence-to-practice gap and position occupational therapists as key actors in planetary health adaptation (43). In summary, OTs can promote Planetary Health in the context of CSHC by addressing the topics of healthy and sustainable lifestyles, health impacts of climate change, and climate and health co-benefits, and by using occupation- and client-centered approaches in situations of routine healthcare activities and situations individually adapted to the client. The integration into routine healthcare activities is also an overarching principle of CSHC, and direct and indirect strategies of CSHC can help to respect the client’s individual values. The CSHC by OTs is enabled and hindered by different opportunities and barriers due to occupational therapy’s framework and professional conditions: In particular, the CHL of OTs needs to be improved, and the ethical conflict needs to be further discussed to overcome the willingness-action gap towards CSHC, to be able to use their existing competencies for CSHC. Networks, movements, and professional associations can support. Overall, CSHC can be an opportunity for OTs to fulfil their role as health professionals in view of the public health relevance of climate change.

The results largely fit into existing findings of CSHC, whereby occupational therapy with its client- and occupation-centered approach seems to have a natural link to CSHC. Nevertheless, to evaluate the effects of CSHC in occupational therapy and thus to be able to assess the actual outcome of CSHC by OTs, intervention studies are needed. The findings of this study can be used to select objectives, topics, settings and situations, approaches and strategies of CSHC in occupational therapy for closer examination. Health psychological models and theories not considered in this study should be included in further studies to investigate the assumed behavioral change effects of CSHC.

### 4.1 Limitations

The data analysis was only conducted by one researcher. The category system was not created and verified in a peer process. In consequence, no intercoder reliability can be assessed (25). But the coding process was revised multiple times by the first author, leading to code saturation and a stable code book (44). Only six interviews were conducted, but this is relativized in view of the presumably small number of existing experts. Due to field access reasons, but still appropriate to the explorative character of this study, participants’ specific knowledge about CSHC was not an inclusion criterion, but special knowledge about planetary health in occupational therapy.

The main challenges in the recruitment process were the partially difficult accessibility of the experts due to their demanding work, and their partially existing uncertainty of being willing to comment on CSHC, as a subject not yet represented in the scientific literature of occupational therapy.

Due to the expert status of the participants, the results can be understood as strong indications of the conditions in the occupational therapy professional groups in the countries. Further empirical research with more OTs actively doing the CSHC in practice is still needed.

## 5 Conclusion

CSHC in occupational therapy offers opportunities to promote the health of people and our planet, with a particular focus on healthy and sustainable lifestyles. The results for France, Germany, and the UK extend the European perspective on CSHC in occupational therapy but cannot be generalized to other countries with different professional structures. Further research, especially outside middle and western Europe, is needed. Additionally, to broaden the current Western scientific perspective on which planetary health is based, future research should not only be conducted in countries that primarily follow this perspective (5, 45). Nevertheless, different perspectives—including the European perspective—as well as international cooperation can promote the further development and implementation of CSHC in occupational therapy.

## Data Availability

The data supporting the findings of this study is available from the corresponding author, AD, upon reasonable requests.

## Footnotes

1 German profession called “Atem-, Sprech- und Stimmlehrerin”.

2 In contrast to the widely known concept of the environmental (or carbon) footprint, its complementary and emerging concept of the environmental handprint comprises the promotion of environmental action to reduce footprints, meaning one’s positive impact (46).
